# Cost-Effectiveness of Real-World Administration of Concomitant *Viscum album* L. Therapy for the Treatment of Stage IV Pancreatic Cancer

**DOI:** 10.1155/2020/3543568

**Published:** 2020-03-12

**Authors:** Anja Thronicke, Thomas Reinhold, Philipp von Trott, Harald Matthes, Friedemann Schad

**Affiliations:** ^1^Research Institute Havelhöhe, Kladower Damm 221, 14089 Berlin, Germany; ^2^Institute of Social Medicine, Epidemiology and Health Economics, Charité—Universitätsmedizin Berlin, Corporate Member of Freie Universität Berlin, Humboldt-Universität zu Berlin, Berlin Institute of Health, 10117 Berlin, Germany; ^3^Oncological and Palliative Care Centre, Hospital Gemeinschaftskrankenhaus Havelhöhe, Kladower Damm 221, 14089 Berlin, Germany; ^4^Medical Clinic for Gastroenterology, Infectiology and Rheumatology CBF, Institute of Social Medicine, Epidemiology and Health Economics, Charité—Universitätsmedizin Berlin, Corporate Member of Freie Universität Berlin, Humboldt-Universität zu Berlin, Berlin Institute of Health, 10117 Berlin, Germany

## Abstract

**Background:**

For patients receiving add-on *Viscum album* L. (VA) treatments for late-stage pancreatic cancer, an improved overall survival (OS) was observed. Only limited information regarding cost-effectiveness (CE) for comparisons between standard of care and standard of care plus add-on VA in stage IV pancreatic cancer treatment is available. The present study assessed the costs and cost-effectiveness of standard of care plus VA (V) compared to standard of care alone (C) for a hospital in Germany.

**Methods:**

An observational study was conducted using data from the Network Oncology clinical registry. Patients included had stage IV pancreatic cancer at diagnosis and received *C* or *V* treatment in a certified German Cancer Center. Cost and cost-effectiveness analyses (CEA) including the analysis of the incremental cost-effectiveness ratios (ICER) were performed from the hospital's perspective based on routine data from the financial controlling department and observed data on OS. The primary result of the analysis was tested for robustness in a bootstrap-based sensitivity analysis.

**Results:**

88 patients (*C* or *n* = 34; *V* treatment in a certified German Cancer Center. Cost and cost-effectiveness analyses (CEA) including the analysis of the incremental cost-effectiveness ratios (ICER) were performed from the hospital's perspective based on routine data from the financial controlling department and observed data on OS. The primary result of the analysis was tested for robustness in a bootstrap-based sensitivity analysis. *n* = 34; *C* or *V* treatment in a certified German Cancer Center. Cost and cost-effectiveness analyses (CEA) including the analysis of the incremental cost-effectiveness ratios (ICER) were performed from the hospital's perspective based on routine data from the financial controlling department and observed data on OS. The primary result of the analysis was tested for robustness in a bootstrap-based sensitivity analysis. *V* treatment in a certified German Cancer Center. Cost and cost-effectiveness analyses (CEA) including the analysis of the incremental cost-effectiveness ratios (ICER) were performed from the hospital's perspective based on routine data from the financial controlling department and observed data on OS. The primary result of the analysis was tested for robustness in a bootstrap-based sensitivity analysis. *C* or *V* treatment in a certified German Cancer Center. Cost and cost-effectiveness analyses (CEA) including the analysis of the incremental cost-effectiveness ratios (ICER) were performed from the hospital's perspective based on routine data from the financial controlling department and observed data on OS. The primary result of the analysis was tested for robustness in a bootstrap-based sensitivity analysis. *C* or *V* treatment in a certified German Cancer Center. Cost and cost-effectiveness analyses (CEA) including the analysis of the incremental cost-effectiveness ratios (ICER) were performed from the hospital's perspective based on routine data from the financial controlling department and observed data on OS. The primary result of the analysis was tested for robustness in a bootstrap-based sensitivity analysis. *C* or

**Conclusion:**

Based on this CEA analysis, from the hospital's point of view, the costs per mean month of OS and per mean hospital stay were lower for patients under combinational standard of care plus VA compared to patients receiving standard of care alone for the treatment of stage IV pancreatic cancer. Further prospective cost-effectiveness studies are mandatory to reevaluate our findings.

## 1. Introduction

Integrative oncology (IO) has been enormously developed and internationally established during the last decades in academic and public cancer centres [1]. Eight of 10 long-term survivors have already utilized IO therapies [2]. As per definition, IO “*is a patient-centered, evidence-informed field of cancer care that utilizes mind and body practices, natural products, and/or lifestyle modifications from different traditions alongside conventional cancer treatments. Integrative oncology aims to optimize health, quality of life, and clinical outcomes across the cancer care continuum and to empower people to prevent cancer and become active participants before, during, and beyond cancer treatment*” [3]. The American Society of Clinical Oncology (ASCO) has recently acknowledged the International Society of Integrative Oncology (SIO) guideline reflecting IO's impact on the improvement of health-related quality of life [4]. Nevertheless, the literature on cost-effectiveness (CE) of IO interventions is limited [5]. Thus, there is a critical need to keep investigating for which patients various IO models might be clinically effective and efficient [5, 6] as detailed analyses of variables determining increased healthcare expenditures are important for hospital systems and decision makers. IO concepts including add-on mistletoe (*Viscum album Loranthaceae*, VA) have been utilized with positive results, usually with high external validity, low adverse events, and high patient satisfaction [7]. Add-on VA treatments for late-stage pancreatic cancer have recently been shown to improve OS [8]. In our recent analysis, we have also reported on advanced and metastasized patient's improved OS receiving IO concepts including add-on VA extracts [9]. Pancreatic cancer ranks fifth position in cancer-related deaths worldwide [10] and fourth position in Germany with a five-year survival rate of only of 9-10% [11]. The majority (approx. 60%) of the patients is diagnosed at an advanced stage [12]. Pancreatic resection with adjuvant chemotherapy (CTx) is the gold-standard approach [13], but in only about 20% of all cases, the tumour is resectable at diagnosis [14]. For palliative treatment, chemotherapy has proven to be superior to best supportive care only [15]. In regards to metastatic pancreatic cancer (mPC), several cost or cost-effective analyses have been conducted, and most of them focussed on specific procedures such as costs of screening methods [16] and standard therapies including resection, radiotherapy, and systemic therapies [17–19]. Cost-effectiveness (CE) analyses are economical evaluations of costs in monetary units (e.g., hospital's costs) and of effectiveness outcomes in nonmonetary units (e.g., saved life years or subjective health-related quality of life). As limited information regarding CE comparisons between standard of care and IO concepts including add-on VA is available and considering the recent efficacy data for add-on VA therapy for mPC, there was an interest in the cost-effectiveness of the combinational therapy compared with the standard of care treatment. Thus, the purpose of the present analysis was to estimate the CE of combinational standard plus add-on VA compared with standard therapy alone in mPC patients from a hospital's perspective.

## 2. Materials and Methods

### 2.1. Study Design, Patients, and Primary Objective

A controlled nonrandomized observational monocentric cohort study was conducted revealing real-world data (RWD) [20] by analysing data from the Network Oncology (NO) clinical registry. The NO is a conjoint clinical register of hospitals, practitioners, and outpatient centres [21]. Patients were included in the analysis who were 18 years or older and who gave written consent, with a histologically proven primary diagnosis of stage IV pancreatic cancer receiving standard of care and surviving more than 21 days. Patients were not included when the death date or the last contact date was not available. Follow-up was performed routinely six months after first diagnosis and annually during the next years. Loss to follow-up was defined as no follow-up visits. The CE analysis took the perspective of the hospital Gemeinschaftskrankenhaus Havelhöhe Berlin (GKHB), at which the patients were treated and which is an Anthroposophic-integrative working hospital harbouring three German Cancer Society (DKG, Deutsche Krebsgesellschaft) certified Organ Centres as a DKG-certified Cancer Centre. The study served as a feasibility study for subsequent IO cost-effectiveness studies. The primary objective of this analysis was to evaluate the CE of VA in addition to standard of care compared to standard of care alone in stage IV pancreatic cancer patients from the hospital's perspective.

### 2.2. Ethics Approval and Consent to Participate

The NO study has been approved by the Ethics Committee of the Medical Association Berlin (Berlin—Ethik-Kommission der Ärztekammer Berlin). The reference number is Eth-27/10. Written informed consent has been obtained from all patients prior to study enrolment. The study complies with the principles laid down in the Declaration of Helsinki.

### 2.3. Data Collection

Structured queries from patient records were run for pancreatic cancer patients (International Classification of Diseases code: C25) using the clinical database NO. Demographic data and hospital-related data (diagnosis, pretreatment, and treatment) were retrieved from the NO. In addition, recorded TNM stages and/or documented metastases were queried with their according date and translated into the Union for International Cancer Control (UICC) stages according to the 8th edition of TNM Classification of Malignant Tumours. UICC stage at first diagnosis was defined as the earliest recorded stage within a month of the diagnosis date. Furthermore, standard oncological treatments were queried with their according date. Application of VA extracts in the context of an IO setting was retrieved with the start and end dates, the application type, and the pharmaceutical used. VA therapy was defined as lasting equal or more than four weeks. Information on hospital stays, length of each hospitalization, and cost weights for each patient indicating disease severity was retrieved from the GKHB hospital's cost-accounting database at the financial controlling department. Any inpatient visit included in the analysis was related to the diagnosis or treatment of the disease. For overall survival outcome analyses, patient's last record including date of death, the last documentation of personal contact, date of interdisciplinary tumour board conferences, or follow-up data were retrieved from the NO registry. Since the cost analyses should reflect the hospital's perspective, inpatient longitudinal cost data were obtained from the GKHB hospital's cost-accounting database according to the German Institute for Hospital Fee Systems (InEK) reporting principles. Cost data included the cost of primary therapy for pancreatic cancer, medication, hospital charges for surgery, anaesthesia, diagnostics, laboratories, professional fees, imaging, normal and intensive care units, and medical and nonmedical infrastructure as incurred. Outpatient costs were not included because they are not captured routinely in the data.

### 2.4. Allocation of Groups

Stage IV pancreatic patients included in the study were classified into one of the two groups: (a) *C* group—patients received only standard of care and no add-on VA therapy and (b) *V* group—patients that received standard of care and add-on VA therapy. Standard of care and add-on VA were applied as per routine clinical care. Nonrandomized allocation to the treatment groups was performed by the physician after elaborate information and patient's decision on treatment options. Applied VA preparations included Abnobaviscum, Helixor, and Iscador VA extracts and were given subcutaneously according to the SmPC [22–24]. Off-label VA application (intravenous and intratumoural) was performed in individual cases.

### 2.5. Statistical Analyses

All statistical analyses were conducted using the software R, version 3.5.2 (2018-12-20 [42]) with the exception of sensitivity analysis which was performed by using MS Excel 2016.

Data are presented using descriptive statistics, normally distributed continuous data by the mean and standard deviation (SD) or 95% CI, and skewed distributions by the median and 95% confidence interval (CI). Binary and categorical variables were presented as absolute and relative frequencies using counts and percentages. For comparison of continuous variables between groups at baseline, the unpaired Student's *t*-test for independent samples was used. For comparison of categorical baseline variables, chi-square analyses were performed. All tests were performed two-sided. *p* values <0.05 were considered as significant.

For the survival outcome, Kaplan–Meier survival and multivariate stratified Cox proportional hazard analyses were calculated as previously reported [25]. The start date for survival analysis was the date of first diagnosis of stage IV pancreatic cancer. Patient survival was calculated from the index date to the patient's last record. Respective data for patient's last record were retrieved from the NO registry. Kaplan–Meier survival was calculated for both groups. For survival analysis including Kaplan–Meier curves and right-censored time-to-event analyses, as well as univariate and multivariate Cox proportional hazard models, the R-package “survival” version 2.41-3 was used, for the implementation of nonparametric estimators for censored event history analysis, the package “prodlim,” version 1.6.1 was used, and to draw survival curves, the package “survminer,” version 0.4.0 was used. Age adjustment of mean OS was performed for both groups using the coxph(surv()) function from the package survival (R). The surv function creates a survival object, which is usually used as a response variable in a model formula. The coxph function fits a Cox proportional hazards regression model incorporating time-dependent variables (here: time as a single survival time/follow-up time value with status indicators: 1 = dead and 0 = alive for possible censoring), time-dependent strata (here: group), and other extensions [26, 27]. To analyse how different factors influence the hazard on patient survival and to reduce potential confounding bias, we employed multivariate stratified Cox proportional hazard model adjusting for age, gender, BMI, comorbidities, and oncological treatment variables including add-on VA treatment. Prior to this analysis, verification analyses were performed whether or not proportional hazard assumptions were met.

Mean number of hospital stays, mean hospitalization length in days, and mean cumulative hospital costs (in €) were calculated for each treatment group and adjusted for significant baseline differences (here: age) using the anova(lm()) function in R to fit a linear model and to carry out analysis of covariance. Cost-associated factors were additionally analysed using multivariable linear regression.

### 2.6. Cost-Effectiveness Analyses

For measuring the cost-effectiveness, data on mean OS, mean number of hospital stays, and results on mean hospitalization length were combined with the results on mean total hospital costs. As a result, the mean hospital costs per month mean OS, the mean hospital costs per mean hospital stay, and the mean hospital costs per mean period of hospitalization were reported.

In the case where the intervention would show a better effect in terms of OS and would be more expensive than the control, the incremental cost-effectiveness ratio (ICER) was calculated. The ICER is defined as a ratio of an additional cost-effectiveness of one intervention (here, *V*) compared to another intervention (here, *C*) and was calculated through dividing the group cost difference by effectiveness difference translating into the mathematical equation ((mean hospital costs *V* − mean hospital costs *C*)/(mean months OS *V* − mean months OS *C*)). To determine to what extent the primary cost-effectiveness results may vary due to many replications, a bootstrap analysis with random 1000-fold resampling population was performed. This analysis accounted for the heterogeneity of hospital's resource consumptions observed in the study. The results of the bootstrap samples were plotted into the four-quadrant diagram (cost-effectiveness plane), which gives graphical information on the results' robustness.

## 3. Results

### 3.1. Patients

**Figure 1:**
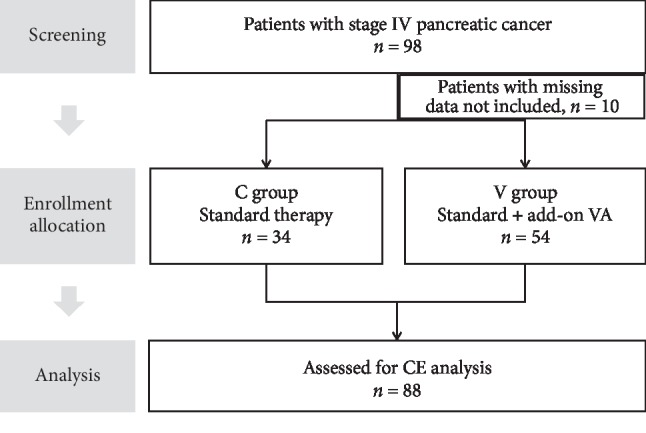
Flowchart of the study population. CE, cost-effectiveness; VA, *Viscum album* L., mistletoe.

From 98 patients who were screened, 10 patients were not included due to missing data, see Figure 1. Thus, 88 patients (*C*: *n* = 34; *V*: *n* = 54) were included for subsequent outcome analysis. Patient's demographic and clinical characteristics are shown in Table 1. The mean age of the patients was 65.6 years. Patients in the *V* group were on average 4.9 years younger than patients in the *C* group (*C*: 68.6 years; *V*: 63.7 years; *p*=0.04). 48.9% of the patients were male. As to gender, BMI, occurrence of comorbidities, arterial hypertension, diabetes mellitus type II, chronic pancreatitis, and pancreatic insufficiency, the patients seem to be largely balanced.

With respect to the total cohort, guideline-oriented surgery (20.5%) and oncological treatment (56.8% gemcitabine as first-line CTx) were applied. In the *V* group, add-on *Viscum album* applications were applied with the majority having received add-on VA from the apple tree (66.7% VA mali), from the ash tree (57.4% VA fraxini), from the oak tree (44.4% VA quercus), and from the fir tree (24.1% VA abietis), see Table 2.

### 3.2. Overall Survival

When patients with *V* treatment are compared to those with *C* treatment, the OS showed a significant increase. Age-adjusted median OS for *C* and *V* was 5.63 (95% CI: 3.37–8.63) and 8.43 months (95% CI: 6.5–12.47), respectively, indicating that patients from the *V* group lived 2.8 months longer than patients from the *C* group (*χ*2 = 7, *p*=0.008). The age-adjusted mean OS for *C* and *V* was 7.1 months and 10.6 months, respectively, and the difference between both groups was 3.5 months, see Table 3. As no death occurred in both groups before the start of the treatment and as time until treatment was not significantly different between both groups (*p*=0.15), a time-dependent bias could be precluded. To analyse how different factors influence the hazard of patient OS and to reduce potential confounding bias, the multivariate stratified Cox proportional hazard model adjusting for age, gender, BMI, comorbidities, and oncological treatment resulted in an adjusted hazard risk (aHR) of 0.35 for chemotherapy indicating a significant 65% reduced risk for the risk of death (aHR: 0.35, 95% CI: 0.20–0.62, *p*=0.0004), while the adjusted hazard risk (aHR) for additional VA therapy was 0.47 indicating a significant 53% reduced hazard of death (aHR: 0.47, 95% CI = 0.28–0.80, *p*=0.005). Furthermore, the direction of impact on hazard of death was negative (i.e., positive for OS) for radiation and surgery but not significant, data not shown. Finally, comorbidities significantly increased the hazard of death by factor 2.1 (aHR: 2.09, 95% CI: 1.13–3.87, *p*=0.02).

### 3.3. Cost Outcomes

**Figure 2:**
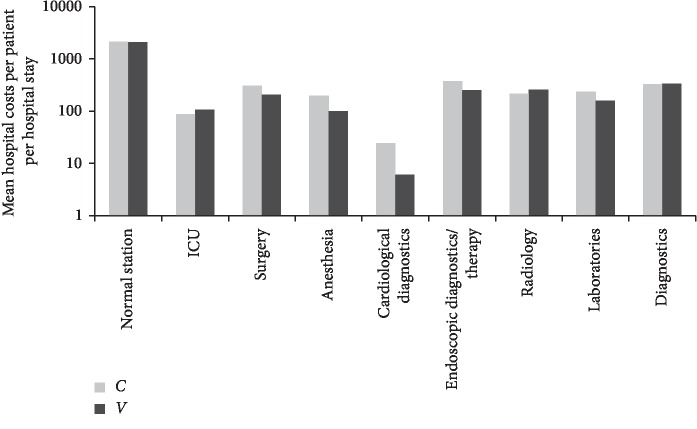
Mean hospital's costs per patient per hospital stay according to the treatment groups *C* and *V* in reliance with the German INEK cost categories, logarithmical y-scale; €.

The cost estimates for both treatment groups are shown in Table 3. Age-adjusted mean hospital's costs were €10069 for the *C* group and €12268 for the *V* group, see Table 3. Highest hospital's expenditures per patient in the *C* group were documented for the normal ward, followed by expenditures for endoscopic diagnostics and therapy, general diagnostics, and surgery, while in the *V* group, the highest expenditures were for the normal ward, followed by expenditures for diagnostics, radiology, and for endoscopic diagnostics and therapy, see Figure 2. Multivariable regression analysis revealed that hospitals costs were highly statistically negatively associated with radiation therapy (estimate −0.25; *p*=0.009). Furthermore, surgery was negatively but not statistically significantly associated with hospital's costs, while the direction of association with age, add-on VA treatment, comorbidities, and chemotherapy was positive but not statistically significant, data not shown.

### 3.4. Cost-Effectiveness

The survival analyses demonstrated that patients in the *V* group showed longer survival than those receiving standard of care (a 3.5 month longer overall mean survival). Cost analyses have shown that this longer survival was associated with additional hospital costs (Table 3). After combining survival data and total costs, the mean costs per mean month OS for the *C* and *V* groups were €1418.16 and €1157.35, respectively. Compared to *C*, patients with *V* treatment had relevant hospital's savings of €260.81 per mean OS, see Table 3. This translates into annual hospital's savings of €3129.72.

We calculated an ICER of €628.28 per additional month OS resembling the costs for the improvement of one OS month gained with the *V* treatment compared to the *C* treatment. This would translate into €7539.32 per additional year OS indicating the costs of improvement of one OS year gained with the new treatment compared to standard of care. Further analyses revealed that (driven by longer survival) patients from the *V* group had on average 1.2 more hospital stays since first diagnosis than patients from the *C* group, see Table 3. Adjusted mean hospital visits in the *C*- and in the *V*-group were 2.21 and 3.37 visits, respectively. As to cost-effectiveness, the mean costs per mean hospital stay for the *C* and *V* patients were €4556.10 and €3640.33, respectively. Consequently, a relevant hospital's saving of €915.77 per mean hospital stay of patients in the *V* group compared to *C* was calculated, see Table 3. Patients from the *V* group had on average a 1.43 day longer hospital stay than *C* treatment patients: the adjusted mean duration of stays for patients from the *V* group was 13.9 days compared to 12.47 days in the *C* group, see Table 3. The calculated mean costs per mean hospitalization day for *C* and *V* were €807.50 and €882.59, respectively, see Table 3. An independent analysis revealed that the median DRG cost weights in the *C* and *V* groups were similar between both groups (*C*: 1.10; *V*: 1.14) indicating similar disease severities, data not shown.

**Figure 3:**
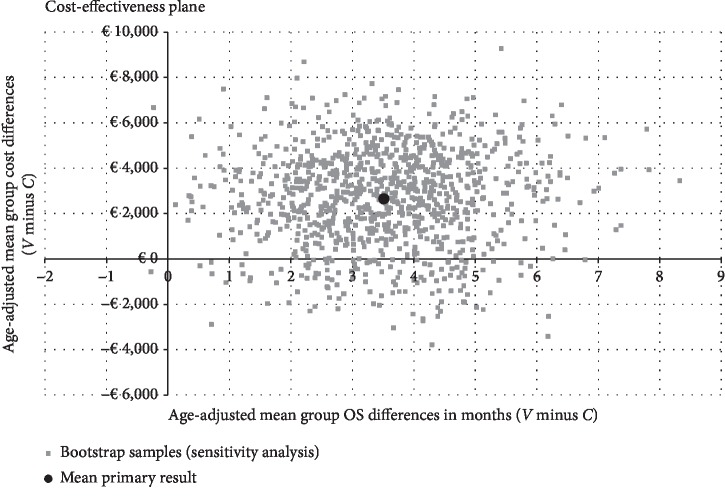
Sensitivity analyses of the outcome cost per month OS from the hospital's perspective. Incremental cost-effectiveness plane showing random 1000-fold resampled estimates (bootstrap analysis) of incremental costs and benefits (age-adjusted mean cost per patient in combination with age-adjusted mean month OS) of using *V* treatment for mPC versus *C* treatment.

For the outcome mean costs per patient from the hospital perspective in combination with mean month OS, a probabilistic sensitivity analysis was performed.  Figure 3 shows a scatter plot of all replicated results for bootstrap sensitivity analyses. Most single dots are located in the upper right-hand quadrant indicating a probability of 87.6% and confirming the robustness of the CEA outcome. Nevertheless, a small proportion of single dots located in the lower right-hand quadrant points to a 12.4% probability of hospital's cost savings.

## 4. Discussion

To our knowledge, this is the first study to directly analyse costs and cost-effectiveness of standard of care plus add-on mistletoe therapy in patients with stage IV pancreatic cancer using patient-level data and actual hospital costing in Germany. From the hospital's point of view, our analysis revealed that compared to standard of care (*C*), patients treated with the combinational therapy (*V*) received a cost-effective therapy with relevant total hospital's savings per mean month OS and per mean hospital stay.

In the present study, we could show that CTx was positively associated with good survival outcome revealing the clinical gold standard for this cancer stage and correlating with data from published studies with CTx's superiority as to OS for advanced or unresectable pancreatic cancer [15, 28]. Next, patients receiving the additional treatment with VA in the present study showed a longer median and mean OS and a significant association with a positive survival outcome. This resembles published clinical outcome results as shown in an RCT and in a real-world analysis for advanced and metastatic pancreatic cancer patients [8, 9]. A relevant negative association of comorbidities including diabetes mellitus type II and chronic pancreatitis with survival outcome was observed in our patients and correlates with other published data [11]. Cost analyses revealed that radiation positively influenced hospital's costs which may be explained by subsequent patient's improvement of tumour status and less hospital's expenditures with treatment efforts. Further cost analyses in the present study indicated somewhat higher costs of the combinational treatment in favourable relation to a better outcome (OS). Higher inpatient hospital costs could either derive from an increased number of hospital stays due to longer OS, from longer hospitalization, from higher DRG cost weights (as an indicator for disease severities), or from a combination of these variables. An independent analysis in the present study revealed that the DRG cost weights were similar between both groups indicating similar and balanced disease severities. Thus, the higher mean costs in the group with the combinational treatment are not explainable by disease severity. Rather, a combination of the slightly higher number of hospital stays due to longer survival, as shown here for the *V* treatment, would serve as an explanation. It has been reported that the coincidence of complementary treatment with increased care durations does not have to be necessarily correlated to a more severe disease status of patients [29]. Given a simultaneous better effectiveness in the present study, combinational therapy with add-on VA has been shown to be noninferior to standard of care alone in terms of cost-effectiveness revealing hospital's savings for this treatment compared to the control. Furthermore, hospital's savings per patient's hospital stay for patients from the *V* group compared to the *C* group were observed, indicating good CEA outcome results. On the contrary, hospitals expenditures per day of each hospitalization were higher in the *V* group compared to the *C* group. This can be explained by the application of several Anthroposophic add-on complementary therapies (beyond add-on VA therapy) [29, 30]. However, compared to earlier studies on pancreatic carcinoma, our data indicate less visits and shorter hospitalization time of the total observed mPC cohort in our study, reflecting current real-life treatment of pancreatic cancer patients as they lie shorter and most of the preparations are increasingly performed in an ambulant setting [31]. Therefore, during further prospective cost-effectiveness studies and by including outpatient cost data in future projects, we will be able to draw a more comprehensive cost spectrum for mPCs. Even though health-related quality of life (HRQL) outcomes in the present study were not assessed, add-on VA's positive impact on HRQL has been repeatedly published [32]. Assuming *V* treatment's noninferiority to *C* treatment in terms of HRQL in mPC patients, hospital's costs of €7539.32 per one year overall survival gained (ICER) by the *V* treatment compared to the *C* treatment are rather low and would thus seem to be affordable in oncological settings.

Limitations of the present analysis include study's retrospective cost analysis, single institution nature, and the limited transferability of cost and CE data to other countries. In addition, through unblinded assignment of add-on VA, physicians could have unintentionally selected patients with better prognosis for this study arm. However, due to known and obvious local skin reactions caused by VA, the procurement of a blinded study seems almost not possible. Furthermore, it has been stated that patients with a healthier lifestyle may be more open for additional complementary therapies and could have selected add-on VA therapy. As sound lifestyle data were not available, this aspect cannot be ruled out so far. Generally, complementary treatment options are frequently enquired by patients in advanced and progressive cancer conditions that could rather result in negative selection bias in the add-on treatment cohort. Further limitations may be the observational nature of the present study, and therefore our findings and conclusions have to be handled with caution. However real-world data as presented here may increasingly contribute to a circular model of evidence [20, 33], complementing published impact of add-on VA in oncology. In future, prospective cost-effectiveness studies with larger patient numbers through application of a multicentre design should limit potential bias. The strength of the present study is its pragmatic design under typical hospital conditions, the inclusion of real-life data, and the model's sensitivity analysis strengthening our observation that the results were comparably robust to the heterogeneity in the underlying patient sample. Thus, the external validity of our data may be high as patient's characteristics, types of treatments, and the effect outcome are compared to published data. This study may state a first onset for a discussion with healthcare administrators and policy makers on the cost-effectiveness of IO concepts for mPC inpatients in German Cancer Centres.

## 5. Conclusions

This is to our knowledge the first extensive evaluation of costs and cost-effectiveness associated with the add-on VA applications in mPC in a German hospital. Based on our findings, the combined standard of care plus VA therapy in these patients seems to be more effective than standard of care only and bears the potential to save hospital costs. In summary, the findings of the present study suggest that the inpatient costs for the combinational *V* therapy compared to *C* come in a favourable relation to a better outcome for stage IV pancreatic patients. Additionally, costs per mean month of OS and per mean hospital stay were lower for *V* compared to *C* treatment. The available data were of observational nature. Further prospective cost-effectiveness studies are mandatory to reevaluate our findings.

## Figures and Tables

**Table 1:** Baseline characteristics of included patients with stage IV mPC.

Variable	All (*n* = 88)	*C* (*n* = 34)	*V* (*n* = 54)	*p* value
Age, mean, SD	65.60 (11.40)	68.61 (10.06)	63.71 (11.87)	0.04
Gender, female, *n* (%)	45 (51.1)	17 (50.0)	28 (51.90)	1
Body mass index <18.5, underweight, *n* (%)	56 (93.3)	20 (90.9)	36 (94.70)	0.97
Comorbidities, yes, *n* (%)	59 (67.0)	21 (61.8)	38 (70.40)	0.55
Arterial hypertension, *n* (%)	32 (36.4)	16 (47.1)	16 (29.6)	0.10
Diabetes mellitus type II, *n* (%)	13 (14.8)	6 (17.6)	7 (13.0)	0.55
Chronic pancreatitis, *n* (%)	2 (2.3)	2 (5.9)	0	0.15
Pancreatic insufficiency, *n* (%)	1 (1.1)	1 (2.9)	0	0.39

*n*, number of patients; %, percent; SD, standard deviation.

**Table 2:** Composition of add-on VA treatment in the *V* group.

	*N* (%)
VA abietis	13 (24.1)
VA aceris	2 (3.7)
VA fraxini	31 (57.4)
VA mali	36 (66.7)
VA pini	5 (9.3)
VA quercus	24 (44.4)

Number of *V* group patients (*n* = 54) exposed to various add-on mistletoe (VA) extracts pooled per host tree. *n* = number of patients; the total number of patients per VA remedy does not necessarily add to 100% as patients may have received various combinations of VA treatment; *N* = number of patients; %, percent.

**Table 3:** Age-adjusted costs and cost-effectiveness measures from hospital's perspective.

	*C*	*V*	Δ_*V*−*C*_
Costs			
Hospital costs^*∗*^, mean €, (95%CI)	10068.97 (6659.87; 13478.06)	12267.94 (9593.67; 14942.20)	2198.97
Outcomes			
OS, median months, (95% CI)	5.63 (3.37; 8.63)	8.43 (6.50; 12.47)	2.8
OS, mean months^*∗*^, (95% CI)	7.1 (4.75; 9.45)	10.6 (8.44; 22.86)	3.5
Hospital stays^*∗*^, mean number, (95% CI)	2.21 (−0.59; 5.01)	3.37 (−1.78; 8.53)	1.16
Hospitalization length^*∗*^, mean days, (95% CI)	12.47 (−0.59–5.01)	13.90 (−1.42; 29.23)	1.43
CE outcomes			
Costs per month OS^*∗*^, mean €/mean months	1418.16	1157.35	−260.81
Costs per hospital stay^*∗*^, mean €/mean number	4556.10	3640.33	−915.77
Costs per days of hospitalization^*∗*^, mean €/mean days	807.50	882.59	75.09

Hospital's costs per mPC patient according to the treatment group; ^*∗*^adjusted for age; €, euro; CI, confidence interval; OS, overall survival; CE, cost-effectiveness; *C*, *C* group; *V*, *V* group; Δ, difference.

## Data Availability

The anonymized data that support the findings of this study are openly available in the repository “figshare” (https://figshare.com/articles/cea/8907338).
